# Evaluation of the efficiency of antibiotics in treating adult patients with symptomatic apical periodontitis: A protocol for systematic review and meta-analysis

**DOI:** 10.1097/MD.0000000000026405

**Published:** 2021-06-25

**Authors:** Wenping Luo, Huifen Yan, Sijie Guo

**Affiliations:** a Department of Stomatology; b Department of Clinical Laboratory, Wuchang Hospital of Wuhan, Wuchang Hospital Affiliated to Wuhan University of Science and Technology, Wuhan, Hubei Province, China.

**Keywords:** antibiotics, apical periodontitis, efficiency, meta

## Abstract

**Background:**

When a person feels dental pain, it brings great discomfort and damages the quality of life. Symptomatic apical periodontitis is identified as the most frequent cause that triggers dental pain. Symptomatic apical periodontitis arises from an infection or inflammation in the pulpless root canal structure. According to clinical guidelines, the primary form of therapy for such teeth entails removing the inflammation or infection source through local surgical procedures. Presently, systemic antibiotics are recommended only for cases where there is clear indication of an infectious spread or a systemic involvement. Therefore, this study aims to assess the efficacy and level of safety of using antibiotics to treat adult symptomatic apical periodontitis patients.

**Methods:**

The present protocol study will conduct a search on electronic databases to look for randomized controlled trials (RCTs) that have evaluated the effectiveness and safety of antibiotics when used to treat adult patients with symptomatic apical periodontitis. The databases will be search from their beginning to April 2021. The search is not bound by publication status or language restrictions. The following databases will be searched: Web of Science, PubMed, the Cochrane Library, Chinese National Knowledge Infrastructure, and EMBASE. This study will employ ZETOC Conference Proceedings and OpenGrey to identify potential grey literature. Afterwards, 2 independent authors will select the studies, extract data from the studies, and conduct a risk assessment to check for bias. All discrepancies between the authors will be resolute via discussion involving a third independent author. The data synthesis and statistical analysis of this study will be done with the RevMan software (Version: 5.3).

**Results:**

The present protocol report will provide high-quality evidence related to the efficacy and level of safety when using antibiotics to treat mature symptomatic apical periodontitis patients.

**Conclusion:**

The outcomes of the present study will update the evidence available for assessing the efficacy and safeness of using antibiotics to treat mature symptomatic apical periodontitis patients.

**Ethics and dissemination:**

This study does not require an ethical approval since individual patient data is not included in any form.

**Registration number:**

DOI 10.17605/OSF.IO/CVP8 M (https://osf.io/cvp8m/).

## 1 Introduction

Apical periodontitis refers to a widespread dental infection centred around the pulpal tissue of the root canal structure. Over time, without proper treatment, an osteolytic apical lesion will form and tooth loss is inevitable.^[1]^ Moreover, latest epidemiologic and mechanistic evidence have established a connection between apical lesion and low-grade general inflammation combined with an elevated risk of cardiovascular disease and diabetes mellitus.^[2–5]^ The origin of symptomatic apical periodontitis could be from a previously healthy tooth that has later suffered pulpal breakdown, or it could arise from a tooth that had asymptomatic apical periodontitis. A dull or excruciating pain that worsens when biting is characteristic of symptomatic apical periodontitis. Generally, the damaged tooth has adverse or delayed positive response to vitality testing, and it also tends to be extremely sensitive to percussive exertions.^[6]^

Prevailing clinical guidelines suggest that primary treatment for teeth showing indications of symptomatic apical periodontitis needs exclusion of the inflammation or infection source via local surgical operations and systemic antibiotics.^[7,8]^ The procedure may require extraction of the offending tooth or extirpation of the pulpal tissues, most likely in combination with the incision and drainage of any prevalent swelling. Presently, systemic antibiotics are recommended only for cases where there are clear symptoms indicating an infectious spread or a systemic involvement.^[7–9]^ Regardless, even in the absence of such symptoms, there is evidence that antibiotics are widely prescribed by dentists to symptomatic apical periodontitis patients.^[10,11]^ However, the level of efficacy in the use of antibiotics remains unclear. Therefore, this study plans to conduct an evaluation of the efficacy and level of safety when using antibiotics as a therapeutic strategy for mature patients with symptomatic apical periodontitis.

## 2 Methods

### 2.1 Study registration

The current protocol of this study has been registered in the OSF (https://osf.io) on April 26, 2021. This study will be conducted in accordance with the Preferred Reporting Items for Systematic Reviews and Meta-Analyses Protocols (PRISMA-P).^[12]^

### 2.2 Criteria for study selection

#### 2.2.1 Types of studies

The current meta-analysis will incorporate each Randomized Controlled Trial (RCT) that has evaluated the efficacy and safety of antibiotics for treating mature symptomatic apical periodontitis patients. The inclusion criteria is not bounded by publication status or language restrictions.

#### 2.2.2 Types of participants

Participants include mature adults (aged 18 years and above) with a clinical diagnosis of symptomatic apical periodontitis in a single tooth.

#### 2.2.3 Types of interventions

It is planned to only include studies using antibiotic (either through intravenous injection or oral administration) as the experimental intervention. Regarding control interventions, this analysis will incorporate additional symptomatic support therapy except antibiotics.

#### 2.2.4 Types of outcome measures

In the present study, the major outcomes include the measures of patient-reported discomfort and swelling, and clinician-reported measures of infection. The minor outcomes include patient-reported quality of life measures, type of analgesics used, analgesic dosage, usage frequency of analgesics, and all adverse effects or harmful outcomes.

### 2.3 Search strategy

A comprehensive search will identify all RCTs that have evaluated the efficacy and safety of using antibiotics to treat mature patients with symptomatic apical periodontitis. The databases will be searched from their inception to April 2021 without any language or publication status restrictions. The following databases will be searched: Web of Science, PubMed, the Cochrane Library, Chinese National Knowledge Infrastructure, and EMBASE. Moreover, this study will employ ZETOC Conference Proceedings and OpenGrey to identify potential grey literature. The search will be conducted through the following combination: apical periodontitis, antibiotics∗, “randomized controlled trials,” and RCT∗.

### 2.4 Data collection and analysis

#### 2.4.1 Selection of studies

**Figure 1:**
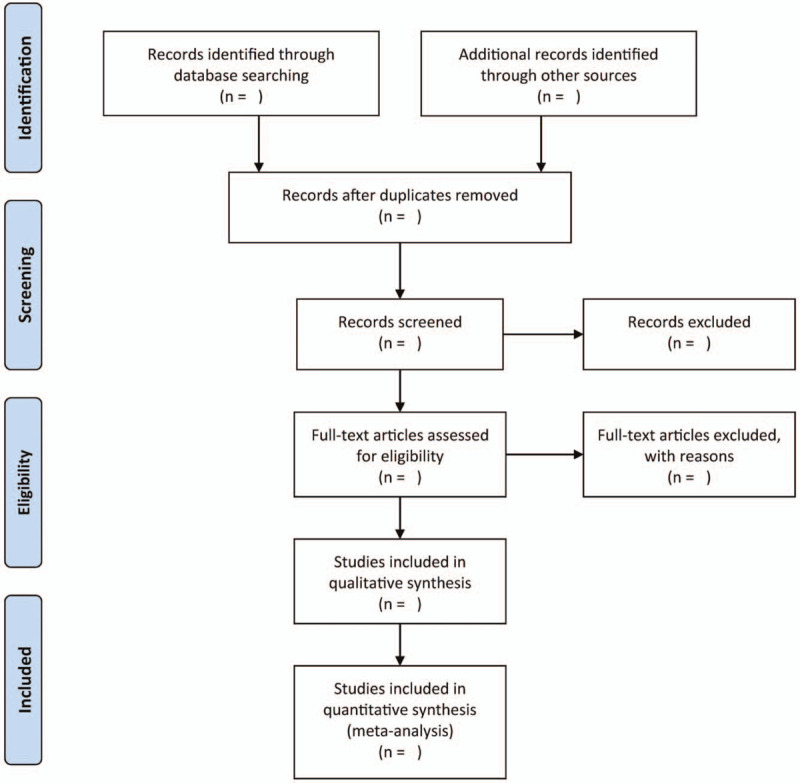
Flow diagram of the literature search.

Firstly, 2 independent authors will perform a title/abstract screening to check if the obtained studies satisfy the inclusion criteria. The full-texts of the studies involved in the first stage will be scrutinized by 2 authors, independent of each other, to satisfy the inclusion criteria. Figure 1 illustrates the study selection flow chart.

#### 2.4.2 Data extraction and management

The study details will be entered into the pilot tested data extraction form. Two independent authors will extract the outcome data from the selected studies with the aid of a generalized data extraction table. All disagreements will be resolved through discussion with a third autonomous author. The following characteristics will be extracted from included studies: participants (sampling setting, criteria for diagnosis, inclusion/exclusion criteria, total number of participants in each group, and baseline demographics), study methodology (designing, distribution, randomization, randomization concealment, blinding, follow-up time, etc), intervention (type of antibiotic, dosage, frequency of use, duration of course, etc), and outcome measures.

#### 2.4.3 Assessment of risk of bias in included studies

A pair of independent authors will evaluate the bias risk of the included studies and find a resolution to all disagreements via discussion with a third independent author. The evaluation of the bias risk in the included studies is based on the Cochrane's Collaboration Tool.

#### 2.4.4 Measures of treatment effect

For dichotomous outcomes, the estimate of effect of the intervention will be expressed as relative risk together with 95% confidence intervals. For continuous outcomes, this study will report mean differences or standardized mean differences together with 95% confidence intervals.

#### 2.4.5 Dealing with missing data

Attempts will be made to establish contact with original authors to fill in instances of missing data.

#### 2.4.6 Assessment of heterogeneity

It is planned to conduct a heterogeneity assessment between the included studies using the *I*^2^ statistic and Chi^2^ test (where, *P* < .1 and *I*^2^ >50% denotes statistical significance).^[13]^ The random-effects model will be utilized to analyze studies judged as statistically significant; otherwise, the fixed-effects model will be utilized.^[14,15]^

#### 2.4.7 Assessment of reporting biases

The probability of publication bias will be evaluated through funnel plots.^[16]^

#### 2.4.8 Sensitivity analysis

The present protocol report will assess the impact of excluding studies with high-risk of bias from the analysis, provided sufficient data were available.

## 3 Discussion

Recently, there has been a steady increase in RCTs evaluating the characteristics of using antibiotics to treat adults with symptomatic apical periodontitis. Admittedly, a considerable number of published studies suggest the application of antibiotics holds a significant position for the treatment of adult patients with symptomatic apical periodontitis. However, the efficacy of using antibiotics to treat grown-ups with symptomatic apical periodontitis is yet to be established. Therefore, we will plan to conduct the present systematic review and meta-analysis to assess the efficacy and safeness of using antibiotics to treat mature patients with symptomatic apical periodontitis. It is hoped that these findings will offer clinicians with the basis for antibiotics of adult patients with symptomatic apical periodontitis.

## Author contributions

**Conceptualization:** Wenping Luo.

**Data curation:** Wenping Luo, Huifen Yan.

**Formal analysis:** Wenping Luo, Sijie Guo.

**Funding acquisition:** Sijie Guo.

**Investigation:** Wenping Luo, Huifen Yan, Sijie Guo.

**Methodology:** Huifen Yan.

**Project administration:** Huifen Yan.

**Resources:** Wenping Luo, Huifen Yan, Sijie Guo.

**Software:** Wenping Luo.

**Validation:** Wenping Luo.

**Visualization:** Wenping Luo, Huifen Yan, Sijie Guo.

**Writing – original draft:** Wenping Luo, Huifen Yan.

**Writing – review & editing:** Sijie Guo.

## References

[1] Virdi RS. Seltzer and Bender's dental pulp, second edition. Br Dental J 2012;213:141-.

[2] Segura-Egea JJ Martín-González J Cabanillas-Balsera D Fouad AF Velasco-Ortega E López-López J. Association between diabetes and the prevalence of radiolucent periapical lesions in root-filled teeth: systematic review and meta-analysis. Clin Oral Investig 2016;20:1133–41. 10.1007/s00784-016-1805-4 27055847

[3] Gupta A Aggarwal V Mehta N Abraham D Singh A. Diabetes mellitus and the healing of periapical lesions in root filled teeth: a systematic review and meta-analysis. Int Endod J 2020;53:1472–84. 32654191 10.1111/iej.13366

[4] Georgiou AC Crielaard W Armenis I de Vries R van der Waal SV. Apical periodontitis is associated with elevated concentrations of inflammatory mediators in peripheral blood: a systematic review and meta-analysis. J Endod 2019;45:1279–95. e3. 31542282 10.1016/j.joen.2019.07.017

[5] Gomes MS Blattner TC Sant’Ana Filho M. Can apical periodontitis modify systemic levels of inflammatory markers? A systematic review and meta-analysis. J Endod 2013;39:1205–17. 24041380 10.1016/j.joen.2013.06.014

[6] Lush V. Textbook of endodontology, 2nd edition. Br Dental J 2010;208:324-.

[7] Lockhart PB Tampi MP Abt E. Evidence-based clinical practice guideline on antibiotic use for the urgent management of pulpal- and periapical-related dental pain and intraoral swelling: a report from the American Dental Association. J Am Dent Assoc 2019;150:906–21. e12. 31668170 10.1016/j.adaj.2019.08.020 PMC8270006

[8] Tampi MP Pilcher L Urquhart O. Antibiotics for the urgent management of symptomatic irreversible pulpitis, symptomatic apical periodontitis, and localized acute apical abscess: systematic review and meta-analysis-a report of the American Dental Association. J Am Dent Assoc 2019;150:e179–216. 31761029 10.1016/j.adaj.2019.09.011 PMC8098651

[9] Palmer N Longman L Randall C. Antimicrobial prescribing for general dental practitioners 2012.

[10] Cope AL Francis NA Wood F Chestnutt IG. Antibiotic prescribing in UK general dental practice: a cross-sectional study. Community Dent Oral Epidemiol 2016;44:145–53. 26507098 10.1111/cdoe.12199

[11] Germack M Sedgley CM Sabbah W Whitten B. Antibiotic use in 2016 by members of the American Association of Endodontists: report of a national survey. J Endod 2017;43:1615–22. 28754406 10.1016/j.joen.2017.05.009

[12] Moher D Shamseer L Clarke M. Preferred reporting items for systematic review and meta-analysis protocols (PRISMA-P) 2015 statement. Syst Rev 2015;4:1. 25554246 10.1186/2046-4053-4-1 PMC4320440

[13] Higgins JP Thompson SG. Quantifying heterogeneity in a meta-analysis. Stat Med 2002;21:1539–58. 12111919 10.1002/sim.1186

[14] DerSimonian R Laird N. Meta-analysis in clinical trials revisited. Contemp Clin Trials 2015;45:139–45. 26343745 10.1016/j.cct.2015.09.002 PMC4639420

[15] Mantel N Haenszel W. Statistical aspects of the analysis of data from retrospective studies of disease. J Natl Cancer Inst 1959;22:719–48. 13655060

[16] Egger M Davey Smith G Schneider M Minder C. Bias in meta-analysis detected by a simple, graphical test. BMJ 1997;315:629–34. 9310563 10.1136/bmj.315.7109.629 PMC2127453

